# Comparative diagnostic utility of metagenomic next‐generation sequencing, GeneXpert, modified Ziehl–Neelsen staining, and culture using cerebrospinal fluid for tuberculous meningitis: A multi‐center, retrospective study in China

**DOI:** 10.1002/jcla.24307

**Published:** 2022-02-24

**Authors:** Yuxin Chen, Yuqing Wang, Xiaojin Liu, Wen Li, Hongyi Fu, Xinyan Liu, Xun Zhang, Xueqin Zhou, Bingzhou Yang, Jie Yao, Xiaolei Ma, Lijun Han, Huan Li, Liheng Zheng

**Affiliations:** ^1^ Department of Laboratory Medicine Nanjing Drum Tower Hospital Nanjing University Medical School Nanjing Jiangsu China; ^2^ Department of Respiratory Medicine No. 4 People’s Hospital of Qinghai Province Xining China; ^3^ 592467 Department of Infectious Disease Hebei Chest Hospital Hebei China; ^4^ 592467 Department of Radiology Hebei Chest Hospital Hebei China; ^5^ 592467 Department of Tuberculosis Hebei Chest Hospital Hebei China; ^6^ 592467 Department of Oncology Hebei Chest Hospital Hebei China; ^7^ 592467 Department of Clinical Laboratory Hebei Chest Hospital Hebei China; ^8^ The Center of Tuberculous Meningitis Diagnosis and Treatment The Infectious Disease Hospital of Changchun Jilin China

**Keywords:** diagnostic efficacy, metagenomic next‐generation sequencing, tuberculous meningitis

## Abstract

**Background:**

Early diagnosis of tuberculosis meningitis (TBM) remains a great challenge during clinical practice. The diagnostic efficacies of cerebrospinal fluid (CSF)‐based mycobacterial growth indicator tube (MGIT) culture, modified Ziehl–Neelsen (ZN) staining, Xpert MTB/RIF, and metagenomic next‐generation sequencing (mNGS) for TBM remained elusive.

**Methods:**

A total of 216 adult patients with suspicious TBM were retrospectively enrolled in this multi‐cohort study. The diagnostic performances for MGIT, modified ZN staining, Xpert MTB/RIF, and mNGS using CSF samples were evaluated.

**Results:**

Uniform clinical case definition classified 88 (40.7%) out of 216 patients as the definite TBM, 5 (2.3%) patients as probable TBM cases, and 24 (11.1%) patients as possible TBM cases. The sensitivities of MGIT, modified ZN staining, Xpert MTB/RIF, and mNGS for TBM diagnosis against consensus uniform case definition for definite TBM were 25.0%, 76.1%, 73.9%, and 84.1%, respectively. Negative predictive values (NPVs) were 66.0%, 85.9%, 84.8%, and 90.1%, respectively. The sensitivities of MGIT, modified ZN staining, Xpert MTB/RIF, and mNGS for TBM diagnosis against consensus uniform case definition for definite, probable, and possible TBM were 18.8%, 57.3%, 55.5%, and 63.2%, respectively. Negative predictive values (NPVs) were 51.0%, 66.4%, 65.6%, and 69.7%, respectively. mNGS combined with modified ZN stain and Xpert could cover TBM cases against a composite microbiological reference standard, yielding 100% specificity and 100% NPV.

**Conclusion:**

Metagenomic next‐generation sequencing detected TBM with higher sensitivity than Xpert, ZN staining and MGIT culture, but mNGS cannot be used as a rule‐out test. mNGS combined with Xpert or modified ZN staining could enhance the sensitivity of diagnostic tests for TBM.

## Graphical abstract

The diagnosis of tuberculosis meningitis (TBM) remains a great challenge during clinical practice. The diagnostic efficacies of cerebrospinal fluid (CSF) Xpert MTB/RIF (Xpert) assay, metagenomic next generation sequence (mNGS), modified Ziehl–Neelsen (ZN) stain, and mycobacterial growth indicator tube (MGIT) culture for diagnosis of TBM were evaluated. mNGS was able to detect TBM with higher sensitivity than Xpert, ZN stain, and MGIT culture. Furthermore, mNGS combined with ZN stain and Xpert could further enhance the sensitivity of diagnosis of clinical suspicious TBM.

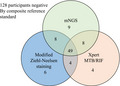

## 1 INTRODUCTION

As a leading cause of morbidity and mortality, tuberculosis affected 10 million people worldwide reported by the World Health Organization (WHO) in 2018.^1^ China is one of high tuberculosis burden countries, which has nearly 8.5% of global estimated tuberculosis incident cases. Tuberculous meningitis (TBM), the central nervous system of tuberculosis, is the most severe manifestation of extrapulmonary tuberculosis, accounting for approximately 1%–5% of all new cases annually.^2^

Tuberculous meningitis arises from a specific bacterium called *Mycobacterium tuberculosis*. Delayed diagnosis and improper treatment might result in poor prognosis and sequel in up to 25% of cases.^3^ Therefore, rapid diagnosis and initiation of treatment is necessary to reduce the high mortality and morbidity associated with the disease.^4^ TBM could be diagnosed by traditional methods using cerebrospinal fluid (CSF) including smear microscopy, mycobacterial growth indicator tube (MGIT) culture, and Ziehl–Neelsen (ZN) staining. Mycobacterial culture remains to be the gold standard for the laboratory of TBM. MGIT culture is a widely used liquid culture system, which is more sensitive to detect low abundance of *M*. *tuberculosis* than the traditional solid media.^5^ Nevertheless, MGIT culture usually takes at least 14 days with moderate sensitivity, which is not readily available in most clinical settings from low‐income countries.^6^ ZN staining to detect acid‐fast bacillus (AFB) is the cheapest and most available test for TBM diagnosis, since it requires minimal specialized equipment and could be rapidly performed. However, the sensitivity of ZN staining is highly dependent on CSF processing steps and microscopist expertise, and the true positive cases are frequently missed.^7^ Modified ZN staining is an efficient and sensitive staining approach which remarkably improved the detection rate of both extracellular and intracellular *M*. *tuberculosis*.^8^ This approach has emerged as a convenient powerful tool for rapid and sensitive diagnosis of *M*. *tuberculosis*.

Over the last decade, emerging technologies also provided promising avenues for TBM diagnosis. Xpert MTB/Rifampicin (RIF) (Cepheid) assay is a rapid, automated, cartridge‐based nucleic acid amplification test, recommended by WHO in 2015 as the initial microbial diagnosis test for TBM.^9^ Xpert MTB/RIF provides 45%–67% sensitivity to detect microbiologically proved TBM, suggesting that a negative result does not provide adequate confidence that TBM is not present.^10^ Subsequently, Xpert MTB/RIF Ultra (Xpert Ultra) was developed with remarkable improved sensitivity over Xpert,^10^, ^11^ with 70% of sensitivity against probable or definite TBM. Meanwhile, metagenomic next‐generation sequencing (mNGS) also emerged as a promising sequencing‐based technique, which has been proven useful to for pathogen identification without a prior knowledge of the target.^12^, ^13^, ^14^, ^15^ Furthermore, mNGS is sensitive to identify any low abundance of microbe infection within a single test, when compared to microbe specific PCR based assays. A recent pilot study from 12 TBM cases revealed a sensitivity of 67% against a reference standard of definite TBM, higher than AFB stain, PCR, and culture.^13^ Therefore, a large, multi‐center study is urgently needed to evaluate clinical utility for these emerging and traditional assays.

Herein, our study aims to retrospectively evaluate the diagnostic performance of MGIT culture, modified ZN staining, Fluid Xpert MTB/RIF, and mNGS using CSF samples among clinical suspected TBM patients. We found that mNGS detected TBM with higher sensitivity than MGIT culture, Xpert, and modified ZN staining. mNGS combined with modified ZN staining or Xpert could enhance the sensitivity of diagnostic tests.

## 2 MATERIALS AND METHODS

### 2.1 Study design

We retrospectively recruited the clinically suspected TBM patients admitted in four large tertiary hospitals during the period from January 2019 to February 2021, including Hebei Chest Hospital, No. 4 people's hospital of Qinghai Province, and Changchun Infectious Diseases Hospital. This study protocol was approved by the Institutional Review Boards of four hospitals. All the patients or their guardians provided the informed written consent prior to research participation. The inclusion criteria for this retrospective cohort were as follows: (i) age over 18; (ii) clinical suspected TBM patients with the classic TBM clinical symptoms and signs including fever, headache, vomiting, and meningeal irritation, and radiographic findings suggesting TBM; (iii) CSF samples were evaluated by MGIT culture, Xpert MTB/RTF, mNGS, or modified ZN staining simultaneously; (iv) the written informed consent was provided. Exclusion criteria were as follows: (i) diagnosis of non‐infectious diseases, and pathogenic infections other than TBM before admission; (ii) refusal of signing the informed consent; (iii) no paired test results for MGIT culture, Xpert MTB/RTF, mNGS, or modified ZN staining.

The recommended clinical diagnosis of TBM involved signs or syndrome considering meningitis, malformed CSF cells (lymphocytic predominance >50%), protein concentration higher than 1 g/L, CSF to plasma‐based glucose ratio <50%, or CSF glucose concentration <2.2 mmol/L and existing TB.^16^ CSF samples were gathered and subsequently subjected to biochemistry analysis, mNGS, Xpert MTB/RIF, modified ZN staining, and MGIT culture.

#### 2.1.1 Microbiological testing

All the patients in this retrospective cohort performed lumbar puncture as a normal procedure for etiology diagnosis of meningitis. CSF samples were collected for simultaneous microbiological analysis of acid‐fast bacillus stain, GeneXpert MTB/RIF, mNGS, and MGIT 960 culture.

#### 2.1.2 MGIT 960 culture

The sterile CSF samples were centrifuged at 3000 *g* for 10 min.

Nonsterile specimens were decontaminated by *N*‐acetyl‐NaOH followed by centrifugation at 4000 *g* for 15 min. The sediment was resuspended in 2 ml phosphate buffer (pH 6.8). 0.5 ml of concentrated CSF specimen was inoculated into MGIT tube containing 0.8 ml of mycobacteria growth indicator tube (MGIT) which was carried out by MGIT 960 system (Becton, Dickinson and Company) according to the manufacturer's instructions and protocols. MGIT tubes were then incubated for a period of 42 days until they were automatically identified as positive by MGIT equipment.

#### 2.1.3 Modified ZN stain

Modified ZN stain was performed as previously described with minor modification.^8^ Cytospin was used to collect the formed elements of CSF specimens. 0.5 ml of CSF specimens was loaded into the chamber in which poly‐l‐lysine‐coated slides were inserted and centrifuged at 28 *g* for 5 min. After aspirating the supernatant, the chambers were removed and the slides were dried before staining with acid‐fast dye according to the standard procedure.^17^ Compared to modified ZN staining described by Chen et al.,^8^ the steps of fixation and permeabilization were removed. The ZN smear was examined using high‐power oil immersion microscopy under 1000× magnification for a minimum of 300 fields. Detection of at least a single acid‐fast bacillus was defined as positive. AFB‐positive fields were counted independently by two experienced technicians.

#### 2.1.4 GeneXpert MTB/RIF

GeneXpert MTB/RIF assay was carried by Xpert MTB/RIF instruments and kits (Cepheid) as per the manufacturer's instruction and protocols. In brief, if the volume of collected CSF specimen was less than 2 ml, the sample reagent was added to CSF specimen to a total volume of 2 ml. Then, the 2 ml of CSF specimen or the mixture of CSF and sample reagent was transferred to the GeneXpert cartridge and loaded onto the Cepheid platform for further analysis.

#### 2.1.5 Metagenomic NGS

Metagenomic NGS was conducted by the BGISEQ‐100 platform for CSF specimens, and sequences of pathogens were compared with *M. tuberculosis* (GenBank ID: GCF_001708265.1). mNGS analysis was performed following three main steps: (i) Processing of sample and extraction of DNA: 600 μl CSF from patients was mixed with equal volume of 0.5‐mm diameter glass beads, vortexed vigorously for 20 min, and centrifuged at 7104 *g* for 1 min. Then, 300 μl supernatant was collected for DNA extraction following the protocols of TIANamp Micro DNA Kit (DP316; Tiangen Biotech) to isolate DNA. Purified DNA was fragmented into 200–300 bp segments using ultrasonic vibration. (ii) Construction of library and sequencing: DNA libraries were constructed through end‐repair, add A‐tailing, adapter ligation and PCR amplification. The DNA libraries were quantified using Qubit dsDNA HS Assay Kit (Thermo Fisher), and its quality was evaluated electrophorectically using Agilent 2100 (Agilent Technologies). Later, DNA libraries were sequenced by BGISEQ‐50/MGISEQ‐2000. (ii) Bioinformatic analysis: low‐quality, low complexity, short sequences (length <35 bp) were first removed to generate clean reads. High‐quality data are aligned to the human reference database (hg19) and Yanhuang genome sequence by Burrows‐Wheeler Alignment (http://bio‐bwa.sourceforge.net/).^18^ Remaining nonhuman sequence reads were aligned to the RefSeq Microbial Genome Database consists of bacteria (6350 species), viruses (4945 species), fungi (1064 species), and parasites (234 species) via the National Center of Biotechnology Information (ftp://ftp.ncbi.nlm.nih.gov/genomes/). The positive results of mNGS were obtained by detecting the unique *M*. *tuberculosis* complex matching sequence (NC_00962.3).

### 2.2 Diagnosis classification of TBM patients

The diagnostic accuracy of mNGS, Xpert MTB/RIF, modified ZN staining, or MGIT culture was evaluated against the consensus uniform research case definition criteria by Marais et al.,^16^ and TBM suspected patients were categorized into definite, probable, possible, or non‐TBM cases. Definite TBM: microbiological confirmed *M*. *tuberculosis* by any of the mNGS, Xpert MTB/RIF, ZN staining, or MGIT culture. Probable TBM: a diagnostic scoring of 12 or beyond was essential with the availability of cerebral imaging, or a diagnostic scoring of 10 or beyond was necessary when cerebral imaging is not available. Possible TBM: a diagnostic scoring from 6 to 11 was essential with the available cerebral imaging, or a diagnostic scoring from 6 to 9 or without cerebral imaging. Non‐TBM: alternative diagnosis with confirmed differential diagnosis and responsive to recorded therapy when anti‐TB treatment was not used.

### 2.3 Statistical analysis

Mann–Whitney U‐test, chi‐squared test, and t‐test were performed for the comparison of demographic data of the TBM and non‐TBM group. For the categorical variables, a chi‐squared test was performed. Sensitivity, specificity, positive predictive value, and negative predictive value (NPV) were carried out by SPSS version 19.0 (IBM). *p* ≤ 0.05 was considered as statistically significant.

## 3 RESULTS

### 3.1 Characteristics of study cohort

**FIGURE 1:**
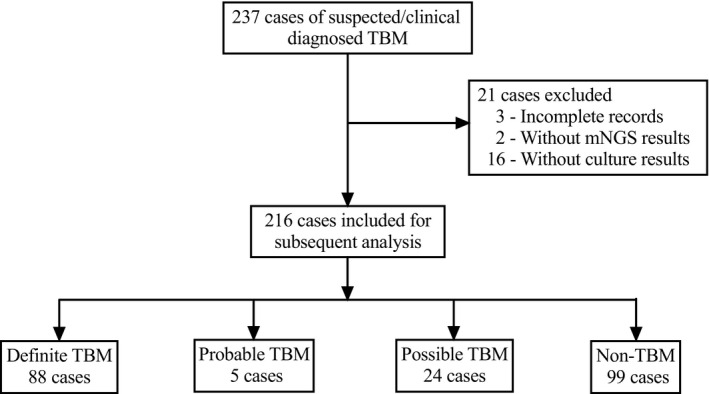
Flow diagram showing the diagnostic outcomes of the study population

Two hundred thirty‐seven suspicious TBM cases were enrolled for evaluating the diagnostic performance of MGIT 960 culture, ZN staining, Xpert MTB/RIF, and mNGS. The demographic and laboratory test characteristics for enrolled patients in this cohort are described in Table 1. Twenty‐one cases were excluded due to the incomplete records (n = 3), the absence of mNGS results (n = 2), and the absence of culture results (n = 16) (Figure 1). Among 216 patients, uniform clinical case definition classified 117 TBM patients as having definite, probable, and possible TBM. Specifically, 88 (40.7%) were confirmed as definite TBM patients due to positive microbiological evidence. Twenty‐two (10.2%) cases were positive for MGIT culture, 67 (31.0%) cases were positive with modified ZN smear, 65 (30.1%) cases were positive with Xpert MTB/RIF, and 74 (34.2%) cases were found with positive mNGS result. Additionally, 5 (2.3%) patients were defined as probable TBM cases, and 24 (11.1%) patients as possible TBM cases. As expected, compared to non‐TBM patients, TBM patients had significantly elevated CSF white cell counts (*p* = 0.000), higher level of CSF protein (*p* = 0.013), decreased CSF glucose (*p* = 0.005), and reduced CSF chloride (*p* = 0.000).

**TABLE 1:** Demographic and laboratory test characteristics in this cohort

Characteristics	All	Definite TBM	Definite, probable TBM	Definite, probable, possible TBM	Non‐TBM	*p* value of TBM vs. non‐TBM
	(n = 216)	(n = 88)	(n = 93)	(n = 117)	(n = 99)	
Age, years	40	40	40	43	39	0.552
	(25–53)	(25–53)	(23–50)	(26–56)	(23–54)	
Gender (male/female)	133/83	54/34	57/36	72/45	61/38	0.991
CSF test						
White blood cells, /μl	153	279	256	218	41	0.000
	(40–332)	(153.5–436.0)	(142–430)	(95–390)	(10–156)	
Protein, mg/dl	1.14	1.48	1.5	1.48	0.82	0.013
	(0.62–1.85)	(1.03–1.90)	(1.04–1.94)	(1.00–2.07)	(0.39–1.57)	
Glucose, mmol/L	2.36	2	2	2.15	2.67	0.005
	(1.68–3.21)	(1.29–2.77)	(1.31–2.8)	(1.41–2.85)	(1.78–3.52)	
Chloride, mmol/L	113.35	110	110	111	115.6	0.000
	(107.95–119)	(104.0–116.0)	(104.00–116.01)	(105.8–166.0)	(109.9–122.9)	

Note

Data are presented as median (interquartile range, IQR).

Abbreviations: CSF, cerebrospinal fluid; TBM, tuberculous meningitis.

### 3.2 Diagnostic performance of individual diagnostic assay for TBM patients

For TBM with definite, probable, and possible patients, MGIT culture showed a sensitivity of 18.8% (95% confidence interval [CI] 12.4–27.3; 22 of 117) and a NPV of 51.0% (95% CI 43.8–58.2; 99 of 194). Among definite and probable TBM patients, MGIT culture yielded a sensitivity of 25.0% (95% CI 16.6–35.6; 22 of 88) and a NPV of 66.0% (95% CI 58.8–72.6; 128 of 194). MGIT culture for definite TBM patients demonstrated relative low sensitivity (25.0%, 95% CI 17.1–35.0; 22 of 88) and a NPV of 66.0% (95% CI 59.1–72.3; 128 of 194).

Modified ZN staining revealed a sensitivity of 57.3% (95% CI 47.8–60.9; 67 of 117) and a NPV of 66.4% (95% CI 58.5–73.8; 99 of 149) for definite, probable, and possible TBM patients. Meanwhile, modified ZN staining revealed 72.0% (95% CI 61.6–80.6; 67 of 93) for sensitivity and 82.5% (95% CI 75.3–88.1; 123 of 149) for NPV among definite and probable TBM patients. Similarly, modified ZN staining showed a sensitivity of 76.1% (95% CI 65.6–84.3; 67 of 88) and a NPV of 85.9% (95% CI 79.0–90.9; 128 of 149) in definite TBM patients.

The diagnostic performance for Xpert MTB/RIF was also evaluated. Xpert MTB/RIF revealed a sensitivity 55.5% (95% CI, 46.1–64.6; 65 of 117) and NPV 65.6% (95% CI, 57.3–73.0; 99 of 151) for definite, probable, and possible patients. For definite TBM and probable TBM patients, Xpert MTB/RIF revealed a sensitivity of 69.9% (95% CI, 59.3–78.7; 65 of 93) and 81.4% NPV (95% CI 74.1–87.1; 123/151). Xpert MTB/RIF yielded a similar 73.9% sensitivity (95% CI 63.2–82.4; 65 of 88) and 84.8% NPV (95% CI 77.8–89.9; 128 of 151) in definite TBM patients.

We then evaluated the diagnostic performance of mNGS in definite, probable, and possible TBM patients, which achieved 63.2% (95% CI 53.8–71.8; 74 of 117) sensitivity and 69.7% NPV (95% CI 61.4–77.0; 99 of 142). Meanwhile, for definite TBM patients combined with probable TBM patients, mNGS approach represented 79.6% (95% CI 69.7–87.0; 74 of 93) for sensitivity and 86.6% (95% CI 79.6–91.5; 123 of 142) for NPV. Among definite TBM patients, mNGS achieved a sensitivity of 84.1% (95% CI 74.4–90.7; 74 of 88) and a NPV of 90.1% (95% CI 83.7–94.3; 128 of 142) (Table 2).

**TABLE 2:** Performance of different methods for diagnosis of tuberculous meningitis

	MGIT culture	Modified ZN staining		Xpert MTB/RIF		mNGS
Reference standard: Definite, probable and possible TBM						
Sensitivity	22/117 (18.8%)		67/117 (57.3%)	65/117 (55.5%)		74/117 (63.2%)
Specificity	99/99 (100%)		99/99 (100%)	99/99 (100%)		99/99 (100%)
PPV	22/22 (100%)		67/67 (100%)	65/65 (100%)		74/74 (100%)
NPV	99/194 (51.0%)		99/149 (66.4%)	99/151 (65.6%)		99/142 (69.7%)
Reference standard: Definite and probable TBM						
Sensitivity	22/93 (23.7%)		67/93 (72.0%)	65/93 (69.9%)		74/93 (79.5%)
Specificity	123/123 (100%)		123/123 (100%)	123/123 (100%)		123/123 (100%)
PPV	22/22 (100%)		67/67 (100%)	65/65 (100%)		74/74 (100%)
NPV	123/194 (63.4%)		123/149 (82.5%)	123/151 (81.4%)		123/142 (86.6%)
Reference standard: Definite TBM						
Sensitivity	22/88 (25.0%)		67/88 (76.1%)		65/88 (73.9%)	74/88 (84.1%)
Specificity	128/128 (100%)		128/128 (100%)		128/128 (100%)	128/128 (100%)
PPV	22/22 (100%)		67/67 (100%)		65/65 (100%)	74/74 (100%)
NPV	128/194 (66.0%)		128/149 (85.9%)		128/151 (84.8%)	128/142 (90.1%)

Abbreviations: MGIT, mycobacterial growth indicator tube; mNGS, metagenomic next‐generation sequencing; NPV, negative predictive value; PPV, positive predictive value; TBM, tuberculous meningitis; ZN, Ziehl–Neelsen.

Our data suggested that the mNGS showed the highest sensitivity and NPV for either definite TBM patients, definite, and probable TBM patients, or definite, probable, and possible TBM patients, when compared to the other three approaches (*p* < 0.005). Modified ZN staining also demonstrated a remarkable sensitivity (>75%) for definite TBM patients, which was superior to Xpert MTB/RIF and MGIT culture (*p* < 0.005). Consistent with previous reports,^8^, ^9^ Xpert MTB/RIF had a reasonable sensitivity for definite TBM patients, whereas the MGIT culture was the least sensitive approach for TBM cases.

### 3.3 Diagnostic performance of combined diagnostic assays for TBM patients

**FIGURE 2:**
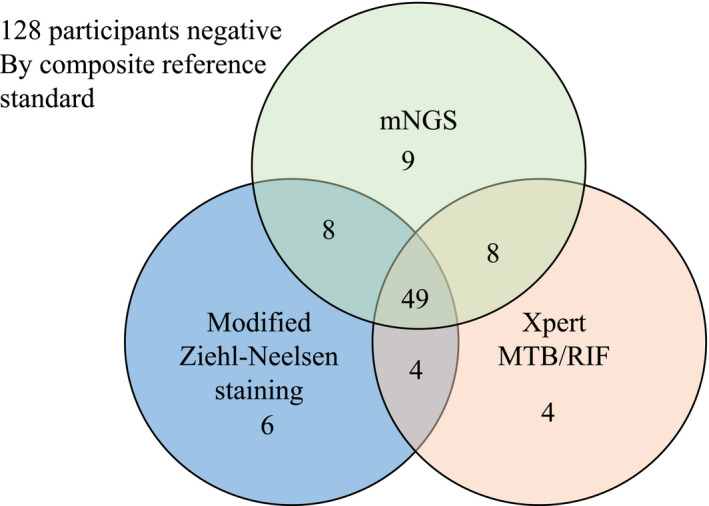
Venn diagram of positive diagnostic tests in the composite microbiological reference standard. The Venn diagram displays 88 participants with microbiological confirmed tuberculous meningitis by either Xpert MTB/RIF, mNGS, or Ziehl–Neelsen staining. mNGS, metagenomic next‐generation sequencing

When considering the distribution and overlap of positive CSF by MGIT, modified ZN staining, Xpert MTB/RIF, and mNGS, Venn diagram of positive diagnostic tests against the composite microbiological reference standard was analyzed. Since all the positive MGIT culture cases were also positive by modified ZN staining, Xpert MTB/RIF, and mNGS, we did not include MGIT culture in this analysis due to its low sensitivity. Herein, we then explored whether combined diagnostic assays could increase the sensitivity and NPV for the definite TBM cases. mNGS detected nine cases of TBM that were not identified by either Xpert or modified ZN staining, Xpert MTB/RIF identified four cases of TBM that were not identified by mNGS or modified ZN staining, while modified ZN staining identified 6 cases of TBM that were not identified by mNGS or Xpert (Figure 2). The combination of modified ZN staining, Mngs, and Xpert could cover all the definite TBM cases, yielding 100% (95% CI 95.0–100, 88 of 88) specificity and 100% NPV (95% CI 96.5–100,128 of 128) (Table 3).

**TABLE 3:** Performance of combined approaches for diagnosis of tuberculous meningitis

	Modified ZN and Xpert	Modified ZN and mNGS	Xpert and mNGS	Modified ZN, Xpert and mNGS
Reference standard: Definite, probable and possible TBM				
Sensitivity	79/117 (67.52%)	84/117 (71.79%)	82/117 (70.09%)	88/117 (75.21%)
Specificity	99/99 (100%)	99/99 (100%)	99/99 (100%)	99/99 (100%)
PPV	79/79 (100%)	84/84 (100%)	82/82 (100%)	88/88 (100%)
NPV	99/137 (72.26%)	99/132 (75.00%)	99/134 (73.88%)	99/128 (77.34%)
Reference standard: Definite and probable TBM				
Sensitivity	79/93 (84.95%)	84/93 (90.32%)	82/93 (88.17%)	88/93 (94.62%)
Specificity	123/123 (100%)	123/123 (100%)	123/123 (100%)	123/123 (100%)
PPV	79/79 (100%)	84/84 (100%)	82/82 (100%)	88/88 (100%)
NPV	123/137 (89.78%)	123/132 (93.18%)	123/134 (91.79%)	123/128 (96.09%)
Reference standard: Definite TBM				
Sensitivity	79/88 (92.05%)	84/88 (95.45%)	82/88 (93.18%)	88/88 (100.00%)
Specificity	128/128 (100%)	128/128 (100%)	128/128 (100%)	128/128 (100%)
PPV	79/79 (100%)	84/84 (100%)	82/82 (100%)	88/88 (100%)
NPV	128/137 (93.43%)	128/132 (96.97%)	128/134 (95.52%)	128/128 (100%)

Abbreviations: MGIT, mycobacterial growth indicator tube; mNGS, metagenomic next‐generation sequencing; NPV, negative predictive value; PPV, positive predictive value; TBM, tuberculous meningitis; ZN, Ziehl–Neelsen.

## 4 DISCUSSION

Diagnosis of TBM is confirmed by detecting *M*. *tuberculosis* bacteria in CSF as a gold‐standard approach. Nevertheless, CSF‐based AFB smear often only showed only a ~10% positive rate, and the culture of *M*. *tuberculosis* represents a 20%–30% positive rate with a duration of 2 weeks.^18^ Thus, rapid and higher sensitivity CSF‐based diagnostic modalities to detect *M*. *tuberculosis* are urgently needed.

In our study, our optimized ZN staining showed optimal sensitivity (57.3%) and NPV (66.4%) for definite, probable, and possible TBM cases, which is significantly higher compared to the conventional ZN staining. We speculate that Cytospin enables to increase the concentration of *M*. *tuberculosis* and facilitate the detection rate of AFB. Additionally, our approach was further modified based on previous report in 2012.^8^ Since most of *M*. *tuberculosis* was extracellularly resided, we removed two unnecessary procedures including fixation with 4% paraformaldehyde and permeabilization using 0.3% TritonX‐100. We speculate such simplification could save additional time and yield higher sensitivity due to several reasons. First, CSF samples were absorbed by the filter paper within Cytospin, and there was no buoyancy during centrifugation. Secondly, the formed elements of CSF specimen were precipitated on a small volume of glass slide to avoid the potential transfer loss. Thirdly, our simplified ZN staining protocol has fewer steps, further reducing the loss of AFB.

Various nucleic acid amplification‐based diagnostic approaches have been developed.^4^, ^19^ Xpert and next‐generation Xpert Ultra assays were evolved to detect *M*. *tuberculosis* from suspicious TBM patients, serving as a rapid diagnostic assay compared to traditional methods.^19^, ^20^ Nevertheless, there are some conflicting data from a recent prospective, randomized, diagnostic accuracy study.^11^ Therefore, new emerging diagnostic modalities for TBM are urgently demanded.

Over the last decade, mNGS approach has been introduced to detect pathogens with complete DNA content as a highly sensitive technology using various types of specimens such as blood, urine, CSF, and sputum.^12^, ^14^, ^21^ A laboratory‐validated clinical mNGS assay for diagnosis of infectious causes of meningitis and encephalitis from CSF yielded 73% sensitivity and 99% specificity compared to the original clinical test results, and 81% positive agreement and 99% negative agreement after discrepancy analysis.^22^ Diverse microbes were identified by mNGS in the CSF of patients with diagnostically challenging meningitis.^23^

Furthermore, several pilot studies using limited samples have demonstrated that mNGS is an alternative method to detect the presence of *M*. *tuberculosis* in CSF samples from clinical suspected TBM patients.^13^, ^24^ A comparative study showed that the mNGS has a significantly higher sensitivity (50.7%) and specificity (85.7%) compared to culture methods in a prospective cohort.^14^ Similarly, our study suggested mNGS achieved a significantly higher sensitivity of 84.1%, compared to either of MGIT culture, Xpert, and modified ZN staining smear against those definite TBM patients. Nevertheless, mNGS was comparatively expensive than the other three tests, which largely limits the routine test and could not identify strain resistance of *M*. *tuberculosis*.

Although mNGS provides a substantial improvement in accurate diagnosis of TBM, it still had a NPV of 90.1% for definite TBM, which does not represent a perfect rule‐out test. Through Venn diagram analysis, the sensitivity of mNGS plus Xpert is higher than that of mNGS plus ZN staining, while the sensitivity of combined mNGS, Xpert, and ZN staining could achieve 100%. Therefore, mNGS combined with additional highly sensitive diagnostic tests might be still necessary in the clinical setting.

Our study also has some limitations. First, the current study was unable to investigate the diagnostic efficacy of Xpert Ultra. Further studies are needed to compare the Xpert Ultra to mNGS in term of TBM diagnosis using CSF samples. Furthermore, we did not include the analysis of the treatment history, which might hamper the sensitivities and efficacies for Xpert and Culture based assay.

## 5 CONCLUSION

To summarize, our study demonstrated that mNGS approach outperforms Xpert assay, ZN staining, and MGIT culture modalities in CSF‐based diagnosis of TBM. Besides, mNGS also can be utilized when it combined with Xpert or traditional diagnostic tests to further improve the sensitive and timely diagnosis of TBM.

## CONFLICT OF INTEREST

The authors declared that they have no potential conflicts of interest.

## AUTHOR CONTRIBUTIONS

The study was designed by Liheng Zheng, Yuqing Wang, and Lujun Han. Experiments were performed by Xiaojin Liu, Huan Li, Wen Li, Hongyi Fu, Xinyan Liu, Xun Zhang, Xueqin Zhou, Bingzhou Yang, Jie Yao, and Xiaolei Ma. And Statistical analysis was performed by Liheng Zheng and Yuxin Chen. The manuscript was written by Yuxin Chen.

## Data Availability

The data that support the findings of this study are available from the corresponding author upon reasonable request.
